# 1-(2,3-Di­methyl­phen­yl)piperazine-1,4-diium tetra­chlorido­cuprate(II)

**DOI:** 10.1107/S1600536813021454

**Published:** 2013-08-10

**Authors:** Safa Ben Mabrouk, Iness Ameur, Sonia Abid, Mohamed Rzaigui

**Affiliations:** a Laboratoire de Chimie des Matériaux, Faculté des Sciences de Bizerte, 7021 Zarzouna Bizerte, Tunisia

## Abstract

In the title salt, (C_12_H_20_N_2_)[CuCl_4_], the Cu^II^ atom occupies a general position in a flattened tetra­hedral environment by Cl ligands, characterized by Cl—Cu—Cl angles of 134.04 (3) and 137.18 (4)°. The six-membered piperazinediium ring adopts a chair conformation. The organic cation and inorganic anion inter­act through N—H⋯Cl and C—H⋯Cl hydrogen bonds, forming a three-dimensional network.

## Related literature

For general background to the properties of tetra­halido­cuprate(II) compounds, see: Solomon *et al.* (1992 ▶); Kim *et al.* (2001 ▶); Panja *et al.* (2005 ▶); Lee *et al.* (2004 ▶); Turnbull *et al.* (2005 ▶); Shapiro *et al.* (2007 ▶). For general background to the geometry of the tetra­halidocuprate(II) species, see: Halvorson *et al.* (1990 ▶). For puckering parameters, see: Cremer & Pople (1975 ▶).
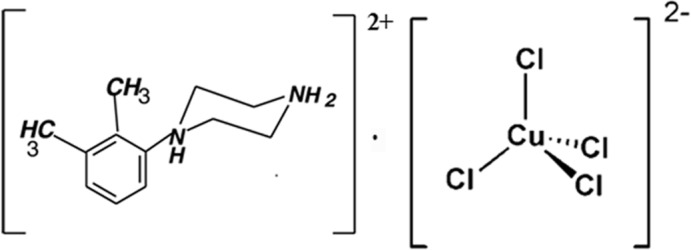


## Experimental

### 

#### Crystal data

(C_12_H_20_N_2_)[CuCl_4_]
*M* *_r_* = 397.64
Triclinic, 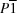

*a* = 7.1986 (15) Å
*b* = 7.7611 (11) Å
*c* = 15.635 (4) Å
α = 77.035 (16)°
β = 79.311 (19)°
γ = 81.845 (14)°
*V* = 831.9 (3) Å^3^
*Z* = 2
Ag *K*α radiation
λ = 0.56087 Å
μ = 1.01 mm^−1^
*T* = 293 K
0.25 × 0.20 × 0.15 mm

#### Data collection

Nonius MACH-3 diffractometer
Absorption correction: part of the refinement model (Δ*F*) (Walker & Stuart, 1983 ▶) *T*_min_ = 0.786, *T*_max_ = 0.863
9228 measured reflections
8079 independent reflections
4600 reflections with *I* > 2σ(*I*)
*R*_int_ = 0.020
2 standard reflections every 120 min intensity decay: 7%

#### Refinement

*R*[*F* ^2^ > 2σ(*F* ^2^)] = 0.050
*wR*(*F* ^2^) = 0.129
*S* = 1.00
8079 reflections
172 parameters
H-atom parameters constrained
Δρ_max_ = 0.87 e Å^−3^
Δρ_min_ = −0.68 e Å^−3^

### 

Data collection: *CAD-4 EXPRESS* (Enraf–Nonius, 1994 ▶); cell refinement: *CAD-4 EXPRESS*; data reduction: *XCAD4* (Harms & Wocadlo, 1996 ▶); program(s) used to solve structure: *SHELXS97* (Sheldrick, 2008 ▶); program(s) used to refine structure: *SHELXL97* (Sheldrick, 2008 ▶); molecular graphics: *ORTEP-3 for Windows* (Farrugia, 2012 ▶); software used to prepare material for publication: *WinGX* (Farrugia, 2012 ▶).

## Supplementary Material

Crystal structure: contains datablock(s) I, New_Global_Publ_Block. DOI: 10.1107/S1600536813021454/ru2053sup1.cif

Structure factors: contains datablock(s) I. DOI: 10.1107/S1600536813021454/ru2053Isup2.hkl

Additional supplementary materials:  crystallographic information; 3D view; checkCIF report

## Figures and Tables

**Table 1:** Hydrogen-bond geometry (Å, °)

*D*—H⋯*A*	*D*—H	H⋯*A*	*D*⋯*A*	*D*—H⋯*A*
N1—H1⋯Cl3^i^	0.91	2.48	3.1610 (18)	132
N2—H2*A*⋯Cl2^ii^	0.90	2.35	3.144 (2)	147
N2—H2*B*⋯Cl1^iii^	0.90	2.30	3.152 (2)	159
N2—H2*B*⋯Cl2^iii^	0.90	2.80	3.271 (2)	114
C2—H2*D*⋯Cl1^i^	0.97	2.74	3.666 (3)	159
C3—H3*B*⋯Cl1^iv^	0.97	2.78	3.585 (2)	141
C4—H4*B*⋯Cl4^ii^	0.97	2.66	3.616 (2)	168
C6—H6⋯Cl4^ii^	0.93	2.71	3.572 (2)	154
C12—H12*C*⋯Cl3^i^	0.96	2.71	3.568 (3)	149

Symmetry codes: (i) 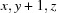; (ii) 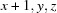; (iii) 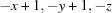; (iv) 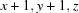.

**Fig. 1.**
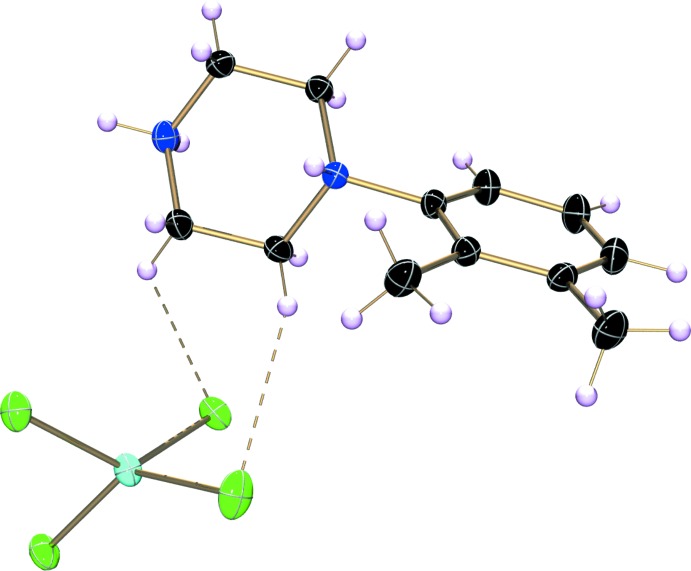
The molecular structure of (I) with 50% probability displacement ellipsoids. Dashed lines indicate C—H···Cl.

**Fig. 2.**
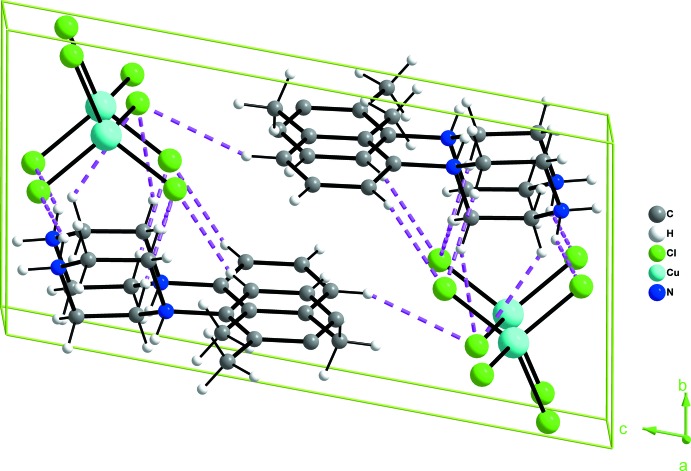
Perspective view of the three-dimensional network of (I), showing the intermolecular hydrogen bonds (dashed solid lines) interactions.

## supplementary crystallographic information

### 1. Comment

Cuprates are chemical compounds in which copper forms complex anions where the overall charge is negative. In such complexes, the ligands are generally cyanides, hydroxides or halides. Due to their important properties, the cuprates still constitute a research axis in many laboratories (Solomon *et al.*, 1992; Kim *et al.*, 2001; Lee *et al.*, 2004; Panja *et al.*, 2005; Turnbull *et al.*, 2005; Shapiro *et al.*, 2007). We report here synthesis and crystal structure of a new cuprate, (C_12_H_20_N_2_)[CuCl_4_] (I). Crystal structure of (I) gives another illustration of this type of material. The asymmetric unit within the unit cell is build of one tetrahedral [CuCl_4_]^2-^ anion and one 1-(2,3-dimethylphenyl)piperazine-1,4-diium cation (Fig. 1). The copper(II) anion exhibits a coordination geometry intermediate between tetrahedral and square–planar. However we can tell that the configuration adopted by this anion is a flattened tetrahedral where the two *trans* bond angles, Cl(1)—Cu—Cl(4) = 137.18 (4)° and Cl(2)—Cu—Cl(3) = 134.04 (3)°, are very near to the minimum of the potential curve describing the angular deformation of isolated [CuCl_4_]^2-^ anion (θ min = 135.95°) (Halvorson *et al.*, 1990). The phenyl ring (C5—C10) of 1-(2,3-dimethylphenyl)piperazine-1,4-diium is planar with an r.m.s. deviation of 0.0111. The 6-membered piperazinium ring adopts a chair conformation, with puckering parameters (Cremer & Pople, 1975) Q_T_ = 0.581 (2) Å, θ = 5.3 (2)° and φ = 328 (3)°. The dihedral angle between the piperazine (N1–N2/C1–C4) ring and the benzene (C5–C10) ring is 65.41 (7) °. In the crystal, neighboring molecules are linked by N—H···Cl and C—H···Cl hydrogen bonds, forming a three-dimensional network (Figure 2).

### 2. Experimental

To an aqueous solution (10 ml) of HCl (0.2*M*) was added 1-(2,3-dimethylphenyl)piperazine (0.19 g, 1 mmol). To the obtained solution, a blue aqueous solution (10 ml) of CuCl_2_.6H_2_O (0.170 g, 1 mmol) was added slowly with stirring. The resulting solution was submitted to a slow evaporation at room temperature until the formation of yellow crystals of the title compound.

### 3. Refinement

H atoms were placed in their calculated positions and then refined using the riding model with atom-H lengths of 0.93 Å (CH), 0.97 Å (CH2), 0.96 Å (CH3), 0.91 Å (NH) and 0.90 Å (NH3). *U*_iso_ were set to 1.2 (CH, CH2), 1.5 (CH3) or 1.20 (NH) times *U*_eq_ of the parent atom.

### Crystal data

**Table:**

(C_12_H_20_N_2_)[CuCl_4_]	*Z* = 2
*M**_r_* = 397.64	*F*(000) = 406
Triclinic, *P*1	*D*_x_ = 1.588 Mg m^−^^3^
Hall symbol: -P 1	Ag *K*α radiation, λ = 0.56087 Å
*a* = 7.1986 (15) Å	Cell parameters from 25 reflections
*b* = 7.7611 (11) Å	θ = 9.0–10.7°
*c* = 15.635 (4) Å	µ = 1.01 mm^−^^1^
α = 77.035 (16)°	*T* = 293 K
β = 79.311 (19)°	Prism, yellow
γ = 81.845 (14)°	0.25 × 0.20 × 0.15 mm
*V* = 831.9 (3) Å^3^	

### Data collection

**Table:**

Nonius MACH-3 diffractometer	4600 reflections with *I* > 2σ(*I*)
Radiation source: fine-focus sealed tube	*R*_int_ = 0.020
Graphite monochromator	θ_max_ = 28.0°, θ_min_ = 2.1°
non–profiled ω scans	*h* = −12→11
Absorption correction: part of the refinement model (Δ*F*) (Walker & Stuart, 1983)	*k* = −12→12
*T*_min_ = 0.786, *T*_max_ = 0.863	*l* = −26→2
9228 measured reflections	2 standard reflections every 120 min
8079 independent reflections	intensity decay: 7%

### Refinement

**Table:**

Refinement on *F*^2^	Primary atom site location: structure-invariant direct methods
Least-squares matrix: full	Secondary atom site location: difference Fourier map
*R*[*F*^2^ > 2σ(*F*^2^)] = 0.050	Hydrogen site location: inferred from neighbouring sites
*wR*(*F*^2^) = 0.129	H-atom parameters constrained
*S* = 1.00	*w* = 1/[σ^2^(*F*_o_^2^) + (0.0614*P*)^2^ + 0.0435*P*] where *P* = (*F*_o_^2^ + 2*F*_c_^2^)/3
8079 reflections	(Δ/σ)_max_ < 0.001
172 parameters	Δρ_max_ = 0.87 e Å^−^^3^
0 restraints	Δρ_min_ = −0.68 e Å^−^^3^
0 constraints	

### Special details

**Table:**

Geometry. All e.s.d.'s (except the e.s.d. in the dihedral angle between two l.s. planes) are estimated using the full covariance matrix. The cell e.s.d.'s are taken into account individually in the estimation of e.s.d.'s in distances, angles and torsion angles; correlations between e.s.d.'s in cell parameters are only used when they are defined by crystal symmetry. An approximate (isotropic) treatment of cell e.s.d.'s is used for estimating e.s.d.'s involving l.s. planes.
Refinement. Refinement of *F*^2^ against ALL reflections. The weighted *R*-factor *wR* and goodness of fit *S* are based on *F*^2^, conventional *R*-factors *R* are based on *F*, with *F* set to zero for negative *F*^2^. The threshold expression of *F*^2^ > σ(*F*^2^) is used only for calculating *R*-factors(gt) *etc*. and is not relevant to the choice of reflections for refinement. *R*-factors based on *F*^2^ are statistically about twice as large as those based on *F*, and *R*- factors based on ALL data will be even larger.

### Fractional atomic coordinates and isotropic or equivalent isotropic displacement parameters (Å^2^)

**Table:**

	*x*	*y*	*z*	*U*_iso_*/*U*_eq_	
Cu1	0.28980 (4)	0.31174 (4)	0.166012 (19)	0.03259 (8)	
Cl3	0.56150 (8)	0.21183 (7)	0.22023 (4)	0.03825 (13)	
Cl2	0.20878 (9)	0.53035 (8)	0.05406 (4)	0.04152 (14)	
Cl1	0.27357 (11)	0.07141 (8)	0.10849 (5)	0.04717 (16)	
Cl4	0.13588 (11)	0.43179 (10)	0.27862 (5)	0.05472 (19)	
N1	0.6470 (2)	0.7932 (2)	0.25996 (11)	0.0256 (3)	
H1	0.5611	0.8853	0.2411	0.031*	
C5	0.6198 (3)	0.7639 (3)	0.35900 (14)	0.0287 (4)	
C10	0.4411 (3)	0.8116 (3)	0.40409 (15)	0.0307 (4)	
C4	0.8414 (3)	0.8412 (3)	0.21292 (15)	0.0298 (4)	
H4A	0.8718	0.9435	0.2317	0.036*	
H4B	0.9365	0.7426	0.2283	0.036*	
N2	0.7973 (3)	0.7293 (3)	0.08360 (13)	0.0342 (4)	
H2A	0.8894	0.6389	0.0928	0.041*	
H2B	0.7938	0.7594	0.0248	0.041*	
C9	0.4213 (3)	0.7840 (3)	0.49714 (16)	0.0342 (5)	
C1	0.6039 (3)	0.6330 (3)	0.23052 (15)	0.0337 (4)	
H1A	0.4785	0.6019	0.2598	0.040*	
H1B	0.6956	0.5328	0.2479	0.040*	
C2	0.6112 (3)	0.6693 (3)	0.13116 (16)	0.0359 (5)	
H2C	0.5914	0.5621	0.1135	0.043*	
H2D	0.5098	0.7603	0.1146	0.043*	
C6	0.7721 (3)	0.6850 (3)	0.40122 (16)	0.0365 (5)	
H6	0.8884	0.6514	0.3688	0.044*	
C3	0.8434 (3)	0.8834 (3)	0.11406 (15)	0.0337 (4)	
H3A	0.7513	0.9847	0.0988	0.040*	
H3B	0.9680	0.9148	0.0839	0.040*	
C7	0.7467 (4)	0.6570 (4)	0.49343 (17)	0.0436 (6)	
H7	0.8462	0.6033	0.5238	0.052*	
C8	0.5736 (4)	0.7093 (3)	0.53963 (17)	0.0420 (5)	
H8	0.5590	0.6937	0.6012	0.050*	
C11	0.2342 (4)	0.8368 (4)	0.55160 (19)	0.0506 (7)	
H70	0.2526	0.8319	0.6114	0.076*	
H72	0.1441	0.7564	0.5520	0.076*	
H71	0.1871	0.9556	0.5260	0.076*	
C12	0.2729 (3)	0.8866 (4)	0.35844 (18)	0.0450 (6)	
H12A	0.1645	0.9099	0.4019	0.067*	
H12B	0.2459	0.8026	0.3269	0.067*	
H12C	0.3005	0.9953	0.3173	0.067*	

### Atomic displacement parameters (Å^2^)

**Table:**

	*U*^11^	*U*^22^	*U*^33^	*U*^12^	*U*^13^	*U*^23^
Cu1	0.03341 (14)	0.03084 (14)	0.03585 (16)	0.00481 (10)	−0.01015 (11)	−0.01292 (11)
Cl3	0.0377 (3)	0.0295 (2)	0.0513 (3)	0.0060 (2)	−0.0183 (2)	−0.0128 (2)
Cl2	0.0450 (3)	0.0395 (3)	0.0405 (3)	0.0100 (2)	−0.0159 (3)	−0.0105 (2)
Cl1	0.0676 (4)	0.0333 (3)	0.0480 (4)	−0.0068 (3)	−0.0224 (3)	−0.0121 (3)
Cl4	0.0545 (4)	0.0622 (4)	0.0416 (3)	0.0228 (3)	−0.0035 (3)	−0.0199 (3)
N1	0.0264 (8)	0.0238 (7)	0.0260 (8)	0.0000 (6)	−0.0036 (6)	−0.0058 (6)
C5	0.0346 (10)	0.0263 (9)	0.0254 (9)	−0.0014 (7)	−0.0033 (8)	−0.0081 (7)
C10	0.0313 (10)	0.0303 (10)	0.0301 (10)	−0.0023 (8)	−0.0025 (8)	−0.0078 (8)
C4	0.0278 (9)	0.0309 (10)	0.0312 (10)	−0.0033 (7)	−0.0045 (8)	−0.0072 (8)
N2	0.0353 (9)	0.0381 (10)	0.0304 (9)	0.0012 (7)	−0.0043 (8)	−0.0129 (8)
C9	0.0373 (11)	0.0330 (10)	0.0309 (11)	−0.0076 (9)	0.0033 (9)	−0.0082 (9)
C1	0.0394 (11)	0.0300 (10)	0.0345 (11)	−0.0097 (8)	−0.0031 (9)	−0.0109 (9)
C2	0.0376 (11)	0.0412 (12)	0.0325 (11)	−0.0069 (9)	−0.0060 (9)	−0.0130 (9)
C6	0.0361 (11)	0.0398 (12)	0.0335 (11)	0.0076 (9)	−0.0078 (9)	−0.0123 (9)
C3	0.0345 (11)	0.0351 (11)	0.0314 (11)	−0.0086 (9)	−0.0004 (9)	−0.0072 (9)
C7	0.0475 (14)	0.0492 (14)	0.0334 (12)	0.0070 (11)	−0.0133 (11)	−0.0090 (11)
C8	0.0550 (15)	0.0419 (13)	0.0290 (11)	−0.0022 (11)	−0.0065 (10)	−0.0092 (10)
C11	0.0481 (15)	0.0613 (17)	0.0385 (14)	−0.0070 (13)	0.0094 (12)	−0.0145 (13)
C12	0.0295 (11)	0.0605 (16)	0.0396 (13)	−0.0013 (11)	−0.0021 (10)	−0.0040 (12)

### Geometric parameters (Å, º)

**Table:**

Cu1—Cl4	2.2170 (9)	C9—C11	1.510 (3)
Cu1—Cl3	2.2439 (8)	C1—C2	1.508 (3)
Cu1—Cl2	2.2467 (8)	C1—H1A	0.9700
Cu1—Cl1	2.2704 (7)	C1—H1B	0.9700
N1—C5	1.493 (3)	C2—H2C	0.9700
N1—C1	1.507 (3)	C2—H2D	0.9700
N1—C4	1.511 (3)	C6—C7	1.390 (3)
N1—H1	0.9100	C6—H6	0.9300
C5—C6	1.382 (3)	C3—H3A	0.9700
C5—C10	1.391 (3)	C3—H3B	0.9700
C10—C9	1.405 (3)	C7—C8	1.376 (4)
C10—C12	1.499 (3)	C7—H7	0.9300
C4—C3	1.504 (3)	C8—H8	0.9300
C4—H4A	0.9700	C11—H70	0.9600
C4—H4B	0.9700	C11—H72	0.9600
N2—C3	1.481 (3)	C11—H71	0.9600
N2—C2	1.488 (3)	C12—H12A	0.9600
N2—H2A	0.9000	C12—H12B	0.9600
N2—H2B	0.9000	C12—H12C	0.9600
C9—C8	1.377 (4)		
			
Cl4—Cu1—Cl3	97.87 (3)	N1—C1—H1B	109.4
Cl4—Cu1—Cl2	98.37 (3)	C2—C1—H1B	109.4
Cl3—Cu1—Cl2	134.04 (3)	H1A—C1—H1B	108.0
Cl4—Cu1—Cl1	137.18 (4)	N2—C2—C1	111.22 (19)
Cl3—Cu1—Cl1	96.67 (3)	N2—C2—H2C	109.4
Cl2—Cu1—Cl1	99.83 (3)	C1—C2—H2C	109.4
C5—N1—C1	111.00 (16)	N2—C2—H2D	109.4
C5—N1—C4	115.08 (16)	C1—C2—H2D	109.4
C1—N1—C4	108.77 (16)	H2C—C2—H2D	108.0
C5—N1—H1	107.2	C5—C6—C7	118.2 (2)
C1—N1—H1	107.2	C5—C6—H6	120.9
C4—N1—H1	107.2	C7—C6—H6	120.9
C6—C5—C10	123.4 (2)	N2—C3—C4	110.92 (18)
C6—C5—N1	118.13 (19)	N2—C3—H3A	109.5
C10—C5—N1	118.40 (19)	C4—C3—H3A	109.5
C5—C10—C9	116.8 (2)	N2—C3—H3B	109.5
C5—C10—C12	123.3 (2)	C4—C3—H3B	109.5
C9—C10—C12	119.9 (2)	H3A—C3—H3B	108.0
C3—C4—N1	109.47 (17)	C8—C7—C6	119.7 (2)
C3—C4—H4A	109.8	C8—C7—H7	120.1
N1—C4—H4A	109.8	C6—C7—H7	120.1
C3—C4—H4B	109.8	C7—C8—C9	121.7 (2)
N1—C4—H4B	109.8	C7—C8—H8	119.1
H4A—C4—H4B	108.2	C9—C8—H8	119.1
C3—N2—C2	111.79 (17)	C9—C11—H70	109.5
C3—N2—H2A	109.3	C9—C11—H72	109.5
C2—N2—H2A	109.3	H70—C11—H72	109.5
C3—N2—H2B	109.3	C9—C11—H71	109.5
C2—N2—H2B	109.3	H70—C11—H71	109.5
H2A—N2—H2B	107.9	H72—C11—H71	109.5
C8—C9—C10	120.1 (2)	C10—C12—H12A	109.5
C8—C9—C11	119.2 (2)	C10—C12—H12B	109.5
C10—C9—C11	120.6 (2)	H12A—C12—H12B	109.5
N1—C1—C2	111.01 (18)	C10—C12—H12C	109.5
N1—C1—H1A	109.4	H12A—C12—H12C	109.5
C2—C1—H1A	109.4	H12B—C12—H12C	109.5
			
C1—N1—C5—C6	88.3 (2)	C12—C10—C9—C11	−2.8 (4)
C4—N1—C5—C6	−35.8 (3)	C5—N1—C1—C2	174.05 (18)
C1—N1—C5—C10	−89.5 (2)	C4—N1—C1—C2	−58.4 (2)
C4—N1—C5—C10	146.48 (19)	C3—N2—C2—C1	−53.9 (3)
C6—C5—C10—C9	3.1 (3)	N1—C1—C2—N2	55.3 (3)
N1—C5—C10—C9	−179.34 (19)	C10—C5—C6—C7	−2.1 (4)
C6—C5—C10—C12	−176.0 (2)	N1—C5—C6—C7	−179.7 (2)
N1—C5—C10—C12	1.6 (3)	C2—N2—C3—C4	56.3 (2)
C5—N1—C4—C3	−174.74 (17)	N1—C4—C3—N2	−59.4 (2)
C1—N1—C4—C3	60.0 (2)	C5—C6—C7—C8	−0.5 (4)
C5—C10—C9—C8	−1.4 (3)	C6—C7—C8—C9	2.1 (4)
C12—C10—C9—C8	177.7 (2)	C10—C9—C8—C7	−1.1 (4)
C5—C10—C9—C11	178.2 (2)	C11—C9—C8—C7	179.3 (3)

### Hydrogen-bond geometry (Å, º)

**Table:**

*D*—H···*A*	*D*—H	H···*A*	*D*···*A*	*D*—H···*A*
N1—H1···Cl3^i^	0.91	2.48	3.1610 (18)	132
N2—H2*A*···Cl2^ii^	0.90	2.35	3.144 (2)	147
N2—H2*B*···Cl1^iii^	0.90	2.30	3.152 (2)	159
N2—H2*B*···Cl2^iii^	0.90	2.80	3.271 (2)	114
C2—H2*D*···Cl1^i^	0.97	2.74	3.666 (3)	159
C3—H3*B*···Cl1^iv^	0.97	2.78	3.585 (2)	141
C4—H4*B*···Cl4^ii^	0.97	2.66	3.616 (2)	168
C6—H6···Cl4^ii^	0.93	2.71	3.572 (2)	154
C12—H12*C*···Cl3^i^	0.96	2.71	3.568 (3)	149

Symmetry codes: (i) *x*, *y*+1, *z*; (ii) *x*+1, *y*, *z*; (iii) −*x*+1, −*y*+1, −*z*; (iv) *x*+1, *y*+1, *z*.

## References

[bb1] Cremer, D. & Pople, J. A. (1975). *J. Am. Chem. Soc.* **97**, 1354–1358.

[bb2] Enraf–Nonius (1994). *CAD-4 EXPRESS* Enraf–Nonius, Delft, The Netherlands.

[bb3] Farrugia, L. J. (2012). *J. Appl. Cryst.* **45**, 849–854.

[bb4] Halvorson, K. E., Patterson, C. & Willett, R. D. (1990). *Acta Cryst.* B**46**, 508–519.

[bb5] Harms, K. & Wocadlo, S. (1996). *XCAD4* University of Marburg, Germany.

[bb6] Kim, Y. J., Kim, S. O., Kim, Y. I. & Choi, S. N. (2001). *Inorg. Chem.* **40**, 4481–4484. 10.1021/ic001396i 11487359

[bb7] Lee, Y. K., Park, S. M., Kang, S. K., Kim, Y. I. & Choi, S. N. (2004). *Bull. Korean Chem. Soc.* **25**, 823–828.

[bb8] Panja, A., Goswami, S., Shaikh, N., Roy, P., Manassero, M., Butcher, R. J. & Banerjee, P. (2005). *Polyhedron*, **24**, 2921–2932.

[bb9] Shapiro, A., Landee, C. P., Turnbull, M. M., Jornet, J., Deumal, M., Novoa, J. J., Robb, M. A. & Lewis, W. (2007). *J. Am. Chem. Soc.* **129**, 952–959. 10.1021/ja066330m 17243832

[bb10] Sheldrick, G. M. (2008). *Acta Cryst.* A**64**, 112–122. 10.1107/S0108767307043930 18156677

[bb11] Solomon, E. I., Baldwin, M. J. & Lowery, M. D. (1992). *Chem. Rev.* **92**, 521–542.

[bb12] Turnbull, M. M., Landee, C. P. & Wells, B. M. (2005). *Coord. Chem. Rev.* **249**, 2567–2576.

[bb13] Walker, N. & Stuart, D. (1983). *Acta Cryst.* A**39**, 158–166.

